# Combined Single Gene Testing and Genome Sequencing as an Effective Diagnostic Approach for Anophthalmia and Microphthalmia Patients

**DOI:** 10.3390/genes14081573

**Published:** 2023-08-01

**Authors:** Rabia Basharat, Kim Rodenburg, María Rodríguez-Hidalgo, Afeefa Jarral, Ehsan Ullah, Jordi Corominas, Christian Gilissen, Syeda Tatheer Zehra, Usman Hameed, Muhammad Ansar, Suzanne E. de Bruijn

**Affiliations:** 1 Department of Biochemistry, Quaid-i-Azam University, Islamabad 45320, Pakistan; 2 Department of Human Genetics, Radboud University Medical Center, 6500 HB Nijmegen, The Netherlands; 3 Department of Neuroscience, Biodonostia Health Research Institute, 20014 Donostia-San Sebastián, Spain; 4 Department of Biotechnology, Mirpur University of Science and Technology (MUST), Mirpur 10250, AJK, Pakistan; 5 Ophthalmic Genetics and Visual Function Branch, National Eye Institute, National Institutes of Health, Bethesda, MD 20892, USA

**Keywords:** anophthalmia, microphthalmia, deep intronic variant, genome sequencing, targeted gene sequencing

## Abstract

Anophthalmia and microphthalmia (A/M) are among the most severe congenital developmental eye disorders. Despite the advancements in genome screening technologies, more than half of A/M patients do not receive a molecular diagnosis. We included seven consanguineous families affected with A/M from Pakistani cohort and an unknown molecular basis. Single gene testing of *FOXE3* was performed, followed by genome sequencing for unsolved probands in order to establish a genetic diagnosis for these families. All seven families were provided with a genetic diagnosis. The identified variants were all homozygous, classified as (likely) pathogenic and present in an A/M-associated gene. Targeted *FOXE3* sequencing revealed two previously reported pathogenic *FOXE3* variants in four families. In the remaining families, genome sequencing revealed a known pathogenic *PXDN* variant, a novel 13bp deletion in *VSX2*, and one novel deep intronic splice variant in *PXDN*. An in vitro splice assay was performed for the *PXDN* splice variant which revealed a severe splicing defect. Our study confirmed the utility of genome sequencing as a diagnostic tool for A/M-affected individuals. Furthermore, the identification of a novel deep intronic pathogenic variant in *PXDN* highlights the role of non-coding variants in A/M-disorders and the value of genome sequencing for the identification of this type of variants.

## 1. Introduction

The development of the eye is a complex process and comprises a coordinated set of events between cells that occurs at the embryonic level, starting from the fourth week of gestation. The major eye structure formation is completed by the seventh week in human embryos [1]. Many genes play a role and are either upregulated or downregulated during these developmental events. These processes are tightly regulated, and any disturbance may lead to malformations. Anophthalmia and microphthalmia (A/M) are ocular defects that could arise during the development of the eye. Microphthalmia is defined as small eyes with axial length <21 mm in adults and <14 mm in newborns [2]. Microphthalmia is mostly observed as a complex phenotype associated with other developmental ocular defects, such as anterior segment dysgenesis, corneal opacity, coloboma, or glaucoma [3]. In contrast, anophthalmia is the complete absence of any eye tissue or any remnant eye structures. The combined prevalence of A/M is 1 in 10,000 births [4].

Genetic factors are considered the most important cause of A/M, but a significant number of studies also highlighted the role of environmental factors, such as viral infection, alcohol, smoking, or drugs [5,6,7]. A/M is a heterogeneous disorder depicting genetic complexity with unknown underlying mechanisms. To date, over 90 genes are known to be associated with syndromic and non-syndromic forms of A/M [8]. Most commonly, pathogenic variants in transcription factors, such as *FOXE3*, *SOX2*, and *VSX2*, are known to cause A/M. Only 20–30% of the patients receive a genetic diagnosis, although diagnostic solve rates are higher in cases with severe bilateral A/M compared to unilateral A/M [9]. A molecular and genetic diagnosis is essential as it significantly influences patient management and is required for better counseling of the affected families. Additionally, resolving the missing heritability involved in A/M and establishing better genotype–phenotype correlations will help in understanding the molecular mechanisms responsible for these disorders. In this study, we evaluated the use of a combined single gene testing and genome sequencing approach to improve the mutation detection rate of A/M affected families.

## 2. Methods

### 2.1. Study Cohort and Clinical Examination

This study was designed according to the principles of the Declaration of Helsinki and was approved by the Institutional Review Board of Quaid-i-Azam University, Islamabad, Pakistan, and the local ethics committee of the Radboud University Medical Center (Nijmegen, The Netherlands). Seven A/M consanguineous families from different regions of Pakistan were included, and informed consent was obtained from all participating individuals or their guardians. The families were included after a thorough clinical examination and family history analysis.

### 2.2. DNA Sequencing

For DNA isolation, 5–8 mL peripheral blood was collected in sterile EDTA vacutainers (BD, Franklin Lakes, NJ, USA). Genomic DNA was extracted using a standard phenol–chloroform extraction method, and DNA concentrations were measured using nanodrop (Titertek Berthold, Pforzheim, Germany). Targeted Sanger sequencing of the *FOXE3* coding region was performed as an initial genetic screening in all the selected families. *FOXE3* was amplified by PCR using HotStarTaq master mix (Qiagen, Germany) following standard procedures; primer sequences are listed in Supplementary Table S1. Because of the high GC-content of *FOXE3*, DMSO (8%) and betaine (0.2 M) were added to the PCR reaction to improve the denaturation of the DNA. The amplified products were purified using Exo-Sap IT (Thermo Fisher Scientific, Waltham, MA, USA) and sequenced at Eurofins Genomics (Louisville, KY, USA). The sequencing data were analyzed using SnapGene v5.2.2 (San Diego, CA, USA).

For *FOXE3*-negative probands, genome sequencing was performed at BGI (Hongkong, China) on a BGISeq500 as described previously with a minimal median coverage of 30-fold per genome [10]. Read-mapping to the Human Reference Genome build GRCh38/hg38 was performed using the Burrows–Wheeler Aligner V.0.78 [11]. Single nucleotide variants (SNV), structural variants (SVs), and copy number variants (CNVs) were called using GATK HaplotypeCaller [12] (Broad Institute, Cambridge, MA, USA), Manta Structural Variant Caller [13], and Canvas Copy Number Variant Caller [14], respectively. All SNVs, SVs, and CNVs were annotated using an in-house pipeline as described previously [10].

### 2.3. Variant Prioritization

Sanger and genome sequencing data were analyzed using SnapGene v.5.2.2 (San Diego, CA, USA) and R-studio v4.1.3 [15], respectively. SNVs (coding and non-coding) were selected based on a minor allele frequency of ≤0.01 in the population database gnomAD (v3.1.2, total population frequency) and the in-house genome database of the Radboudumc (containing ~1400 alleles). All rare variants were assessed in detail, and nonsense, frameshift, start loss, start gain, in-frame deletions or insertions, missense, and potential splice-altering variants were prioritized. A potential effect of missense variants was investigated using the in silico tools CADD-PHRED (≥15) [16] and REVEL (≥0.3) [17]. Potential splice site variants were selected based on the deep learning splice predicting tool SpliceAI (delta score ≥ 0.2, default settings) [18]. SVs and CNVs were selected based on a minor allele frequency of ≤0.01 in the population database 1000 Genomes (1000 G) [19]. Coding SVs and CNVs were prioritized when overlapping with (deletions) or at least one of the breakpoints (duplications, inversions, and translocations) located in a protein-coding region. All compound heterozygous and homozygous variants based on SNV, CNV and SV analysis were selected. A manually curated list of 147 (candidate) syndromic and non-syndromic A/M-associated genes was generated using MIM disease terms (OMIM [20], assessed 1 December 2022) (Supplementary Table S2). All prioritized homozygous and compound heterozygous variants overlapping with an A/M-associated gene were selected for validation and segregation analysis using Sanger sequencing (primers listed in Supplementary Table S1). The validated candidate variants were classified according to the American College of Medical Genetics and Genomics and Association of Molecular Pathology (ACMG/AMP) classification system, as described in [21].

### 2.4. In Vitro Minigene Splice Assay

The potential splice-altering effect of a deep intronic *PXDN* (NM_012293.3) variant located in intron 17 was assessed using a minigene splice assay. A 693bp region of intron 17 was amplified using Q5 high-fidelity polymerase (New England BioLabs, Ipswich, MA, USA) from genomic DNA obtained from individuals V:4 and III:1 (family MA144). The primers for amplification were designed with attB1 and attB2 tags at the 5′ ends to allow for Gateway^®^ cloning. The amplified region was first cloned in a pDONR^TM^201 vector (Invitrogen, Waltham, MA, USA) and subsequently into a vector containing *RHO* exons 3 and 5 (pCI-neo) using Gateway^®^ cloning technology (Thermo Fisher Scientific, Carlsbad, CA, USA). Genomic inserts of the created pDONR vectors were verified using single molecule real-time sequencing (Pacific Biosciences, Menlo Park, CA, USA) on a Sequel II system, as described in [22]. Both mutant and wildtype clones were transfected into HEK293T cells using polyethylenimine (PEI) as a transfection reagent. After 24 h of incubation, RNA was extracted, and cDNA was synthesized as previously published [23]. RT-PCR was performed to observe different splice isoforms using primers located in *RHO* exon 3 and *RHO* exon 5 (Supplementary Table S1). The observed splice products were purified from agarose gel and analyzed using Sanger sequencing (ABI3730XL platform, Thermo Fisher Scientific, Waltham, MA, USA).

## 3. Results

### 3.1. Clinical Evaluation of All Affected Members

**Figure 1:**
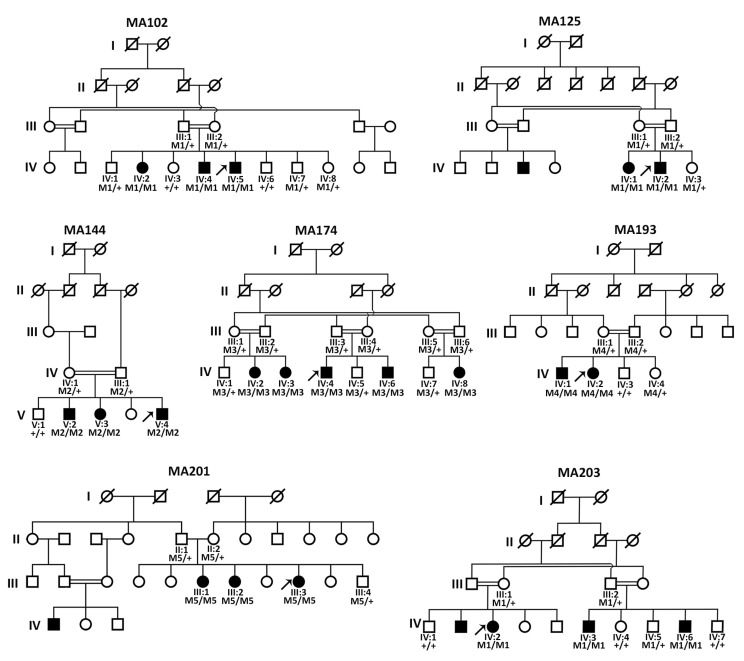
Pedigrees of seven consanguineous families affected with anophthalmia or microphthalmia. Pedigree numbers and segregation analysis results are indicated for all subjects that participated in the study. The proband of each family is indicated with an arrow. M1, *FOXE3* (NM_012186.3): c.720C>A; p.(Cys240*); M2, *PXDN* (NM_012293.3): c.3609-1307G>A; p.Arg1203Serfs76*; M3, *VSX2* (NM_182894.3): c.413_425del; p.(Ser138*); M4, *PXDN* (NM_182894.3): c.2568_2568delC; p.(Cys857Alafs*5); M5, *FOXE3* (NM_012186.3): c.289A>G; p.(Ile97Val); +, wildtype.

**Figure 2:**
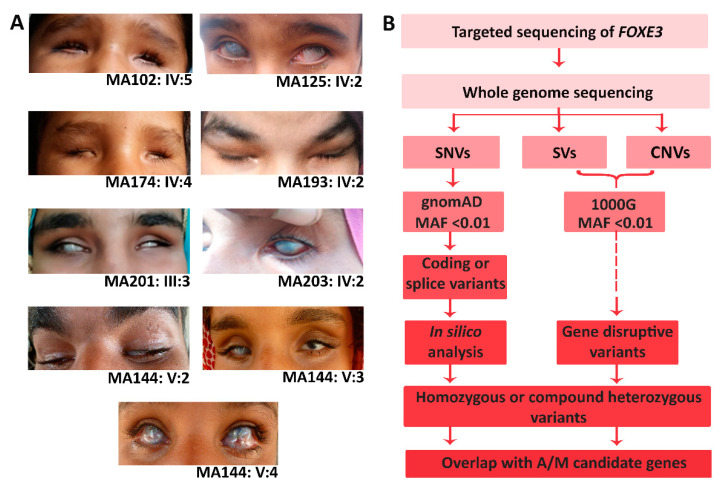
Clinical images and genetic testing workflow. (**A**) Eye images of one affected individual representing each family are provided. For family MA144, images for all three affected members are given, illustrating the intra-familial heterogeneity. (**B**) As an initial screening, targeted Sanger sequencing of *FOXE3* was performed for all unsolved families. Subsequently, genome sequencing was performed for the remaining unsolved families. Single nucleotide variants (SNVs), structural variants (SVs), and copy number variants (CNVs) were prioritized based on a minor allele frequency (MAF) of <0.01 (for SNVs: gnomAD v.3.1.2. and an in-house genome database of Radboudumc (containing ~1400 alleles), SVs and CNVs: 1000 Genomes (1000G)). All coding variants or potential splice-altering variants were selected and assessed using in silico prediction tools (splice-AI, (≥0.2; default settings), CADD-PHRED (≥15), and REVEL (≥0.3)). Additionally, all coding SVs and CNVs overlapping with A/M-associated genes were interrogated in detail and gene-disruptive variants were prioritized. All compound heterozygous or homozygous variants overlapping with an A/M-associated gene (OMIM) were selected for validation and segregation analysis.

A total of 7 families with 20 affected individuals showing A/M with or without corneal opacity or anterior segment dysgenesis were included in this study. All seven Pakistani origin families were consanguineous and showed an autosomal recessive inheritance of the A/M phenotype (Figure 1). All affected individuals were diagnosed with bilateral, congenital microphthalmia (*n* = 10) or anophthalmia (*n* = 10). Detailed clinical features for all individuals are provided in Table 1, and clinical images of the proband of each family are shown in Figure 2.

### 3.2. Sanger Sequencing Revealed FOXE3 Pathogenic Variants in Four Families

**Figure 3:**
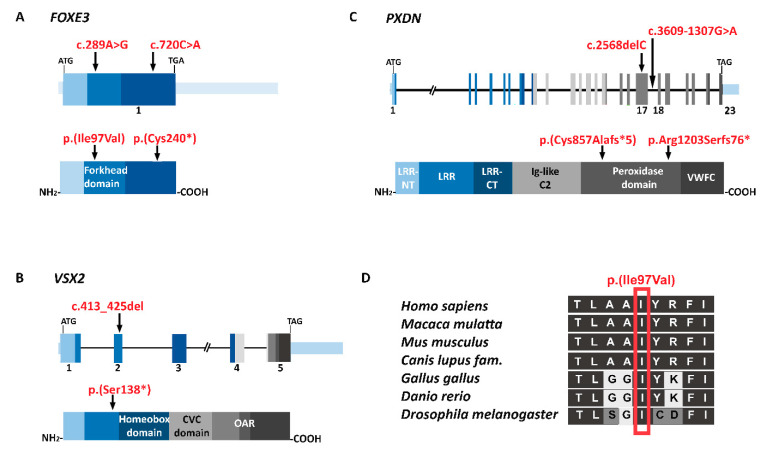
Graphical representation of identified pathogenic variants and affected genes and protein domains. (**A**–**C**) Potentially pathogenic homozygous variants were identified in *FOXE3*, *PXDN*, and *VSX2*. For each variant, the predicted effect on gene (**top**) and protein (**bottom**) level has been indicated. For each gene, relevant exon numbers and variant position are provided. Protein domains encoded by the different exons have been determined using UniProt (accessed January 2023). (**D**) The *FOXE3* missense variant (c.289A>G, p.(Ile97Val)) that was identified in family MA201 affects an evolutionary conserved amino acid (Source: pBLAST). The mutated amino acid is indicated with a red box. CVC, CVC domain; Ig-like C2, C2-type Ig-like domain; LRR, leucine-rich repeats; LRRNT, Leucine-rich repeats N-terminal; LRRCT, Leucine-rich repeats C-terminal, OAR, Otp, aristaless and rax domain; VWFC, Von Willebrand factor C-type domain.

Since the phenotype of 60% of A/M families from a similar Pakistani cohort could be previously explained by pathogenic *FOXE3* variants [24], targeted Sanger sequencing of *FOXE3* was performed as an initial genetic test for all families. In the probands of families MA102, MA125, MA201, and MA203, potentially pathogenic biallelic variants were identified, which segregated with the A/M-phenotype (Figure 1, Supplementary Figure S1). For three of these families, MA102, MA125, and MA203, an identical homozygous nonsense variant (NM_012186.3:c.720C>A; p.(Cys240*)) was identified with an allele frequency of 0.00001334 in gnomAD and only in a heterozygous state. The variant most likely results in the formation of a truncated FOXE3 protein. The variant has been previously reported as pathogenic in ClinVar [25] and classified as pathogenic according to the ACMG/AMP classification system. In family MA201, a known [24] *FOXE3* homozygous pathogenic missense variant (c.289A>G; p.(Ile97Val)) was identified. This amino acid change affects an evolutionary conserved region (up to *Drosophila melanogaster*) of the protein (Figure 3) and is predicted as pathogenic by both REVEL and CADD_PHRED prediction tools (Table 2). No other rare variants were identified in *FOXE3* in any of the investigated probands.

### 3.3. Genome Sequencing Revealed Pathogenic Variants in PXDN and VSX2

The phenotype of three families (MA144, MA174, and MA193) remained genetically unsolved after targeted sequencing of *FOXE3.* Therefore, the probands MA144-(V:4), MA174-(IV:4), and MA193-(IV:2) were subjected to genome sequencing. Variant prioritization revealed possibly pathogenic variants in the A/M-associated genes *PXDN* and *VSX2*, which were also segregated in the respective families (Figure 2, Supplementary Figure S1). In family MA174, a 13bp deletion in *VSX2* (NM_182894.3:c.413_425del; p.(Ser138*)) was identified. Pathogenic variants in *VSX2* are associated with isolated microphthalmia and microphthalmia with coloboma [28]. This frameshift variant has not been previously reported, and results in the formation of a premature termination codon in exon 2 and may induce nonsense-mediated decay (NMD) or the formation of a truncated protein. In family MA193, a known pathogenic [27] single nucleotide deletion (NM_012293.3:c.2568del;p.(Cys857Alafs*5)) was identified in *PXDN*. Both the identified *VSX2* and *PXDN* frameshift variants were classified as pathogenic according to the ACMG/AMP classification system.

In family MA144, a novel homozygous deep intronic variant in intron 17 of *PXDN* (NC_000002.12:g.1646059C>T (c.3609-1307G>A; p.(?)) was identified. The potential splice-altering effect was predicted by SpliceAI (acceptor gain, 0.97) and other prediction tools embedded in the Alamut Visual software version 1.4 (Interactive Biosoftware, Rouen, France; http://www.interactive-biosoftware.com, accessed on 8 July 2023, including MaxEntScan, SpliceSiteFinder-like, NNSPLICE, and GeneSplicer. Based on the high prediction scores of SpliceAI (0.97), SpliceSiteFinder-like (88.9), MaxEntScan (8.5), NNSPLICE (1.0), and GeneSplicer (9.9), the identified splice site variant was predicted to create a strong splice acceptor site, potentially leading to the activation of a pseudoexon. No other homozygous or compound heterozygous candidate variants (SNVs, CNVs, or SVs) in A/M-associated genes were identified in any of the probands.

### 3.4. Pseudoexon Activation in PXDN Caused by a Deep Intronic Splice Variant

**Figure 4:**
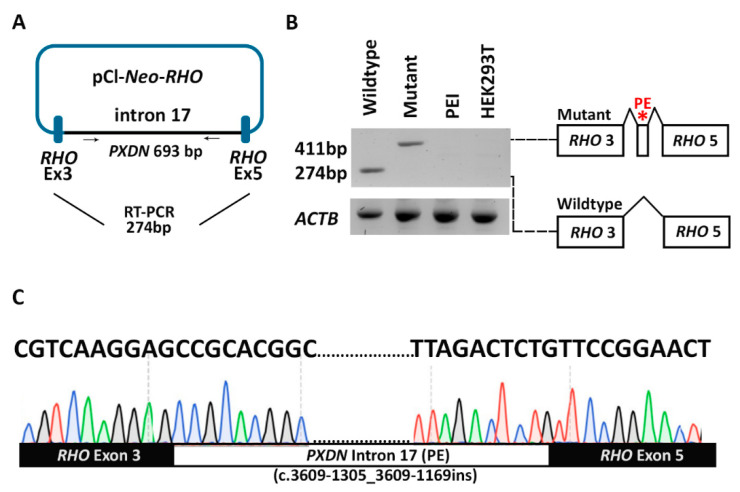
Results of the minigene splice assay for the *PXDN* c.3609-1307G>A deep intronic variant. A minigene splice assay was performed in HEK293T cells to validate the effect of a deep intronic splice variant identified in *PXDN*. The assay confirmed the activation of a 137bp out-of-frame pseudoexon (PE) (c.3609-1305_3609-1169ins; p.Arg1203Serfs76*) in intron 17 of *PXDN* as predicted by several in silico tools, such as the SpliceAI, SpliceSiteFinder-like, MaxEntScan, NNSPLICE, and GeneSplicer. (**A**) Schematic illustration of the minigene construct. (**B**) Gel image showing wildtype (*RHO* exon 3–*RHO* exon 5) and mutant (*RHO* exon 3–pseudoexon (PE)–*RHO* exon 5) products amplified using *RHO* exon 3 and *RHO* exon 5 primers. (**C**) Sequencing chromatogram confirming the pseudoexon insertion in intron 17 of *PXDN.* Wildtype, HEK293T cells transfected with a wildtype *PXDN* construct; mutant, HEK293T cells transfected with a *PXDN* construct harboring the c.3609-1307G>A deep intronic variant; PEI, transfection reagent-only; HEK293T, untransfected HEK293T cells.

To investigate the potential splice effect of the *PXDN* deep intronic variant (c.3609-1307G>A), identified in family MA144, an in vitro minigene splice assay was performed. The variant was present in intron 17, and SpliceAI predicted a 137bp pseudoexon insertion in this intron resulting from this variant (Figure 4). The splice assay confirmed the pseudoexon activation in intron 17 (c.3609-1305_3609-1169) in *PXDN*, which matches the predictions of SpliceAI, and other splicing tools, as discussed earlier (Figure 4). This out-of-frame pseudoexon inclusion results in the formation of a premature termination codon and will most likely cause either NMD or the formation of a truncated PXDN protein (p.Arg1203Serfs76*) that lacks the essential peroxidase domain of peroxidasin homolog protein (Figure 3). No wildtype transcript could be observed when transfection was performed with the mutant construct, suggesting that the variant has a severe, complete, effect on splicing. Based on the splice assay results and confirmed splice-altering effect for the *PXDN* variant, the variant is classified as likely pathogenic according to ACMG/AMP guidelines [21].

## 4. Discussion

A/M is a group of structural ocular defects with varying degrees of severity, ranging from unilateral to bilateral and from simplex (non-syndromic) to complex (syndromic) types. A/M is a complicated disorder, and the underlying mechanisms of the disease are still poorly understood. Some suggest that A/M disorders arise due to secondary regression during ocular development [29], while others hypothesize that these are the results of either lens induction failure [30] or disruptions in optical invagination or during early differentiation of the retina [31,32]. Although environmental factors can contribute to the development of A/M, genetic factors are suggested to be the most common cause of A/M. There are >90 genes reported to be associated with A/M, and yet the phenotype of a significant number of cases cannot be genetically explained [8]. Resolving the missing heritability for A/M is essential, as it is required not only to allow optimal genetic diagnostics, presymptomatic screening in case of a syndromic phenotype, disease management, and genetic counselling of the families, but also to investigate genotype–phenotype correlations which would allow a better understanding of this severe congenital disorder. In the current study, we have genetically explained the phenotype of 20 affected A/M individuals from 7 unrelated Pakistani consanguineous families and investigated their genotype–phenotype correlations. Both novel and previously reported pathogenic variants were identified as affecting three different genes: *FOXE3*, *PXDN*, and *VSX2*.

A previously investigated cohort of the same origin (*n* = 8) suggested that the majority of Pakistani A/M-affected families were explained by *FOXE3* pathogenic variants (60%) [24]. Similarly, in a mixed cohort of Caucasians, Hispanics, African Americans, and Asians (*n* = 116), 15% of the bilateral microphthalmia patients were solved with *FOXE3* variants [33]. This prompted us to first screen this single exon gene that encodes the *FOXE3* transcription factor in our cohort using Sanger sequencing. The screening of *FOXE3* gene solved 57% (4/7) of our families. Although based on a relatively small cohort, this suggests that pre-screening of *FOXE3* prior to any NGS application in Pakistani A/M patients, and possibly for other ethnicities as well, is cost-effective. We have identified two known pathogenic variants in *FOXE3* in four different families. One known homozygous missense (c.289A>G; p.(Ile97Val)) variant was identified in family MA201 that affects an evolutionarily conserved amino acid in the DNA-binding forkhead domain of the protein [24]. Hence, it will most likely affect the DNA-binding affinity of this transcription factor. A second disease-causing *FOXE3* variant (c.720C>A; p.(Cys240*)) was identified in three of the studied families (MA102, MA125, and MA203). This variant was initially reported by Valleix et al. in 2006 in affected members of a Madagascar inbred family [26]. Later, it was identified in Bangladeshi, Kuwaiti, and Pakistani families as well [33,34,35]. This suggests that this variant could be a founder variant inherited from a common ancestor. The known pathogenic variants previously identified in *FOXE3* are predominantly responsible for causing aphakia, sclerocornea, microphthalmia, anterior segment dysgenesis, and, rarely, increased intraocular pressure, bilateral congenital cataract, and vitreoretinal dysplasia [24,34,36,37]. In family MA201, we observed microphthalmia with corneal opacity. Patients from families MA125 and MA203 showed similar phenotypes, including bilateral microphthalmia, corneal opacity and anterior segment dysgenesis. In comparison to this, affected individuals of family MA102 harboring the same *FOXE3* variant showed complete anophthalmia. They also have a flat nasal bridge, but no other facial dysmorphism was observed. This indicates that some genetic or environmental modifiers might play a role.

Using genome sequencing, pathogenic variants in *PXDN* and *VSX2* were identified. *VSX2* encodes the VSX2 retina-specific transcription factor that is highly expressed during embryonic and fetal eye development [38]. *VSX2* pathogenic variants are found in 2% of A/M cases [2]. We identified a novel 13bp deletion in exon 2 of this gene in affected members of family MA174 that presented with complete bilateral anophthalmia. The majority of pathogenic variants reported in *VSX2* cause loss of function either by NMD or by the formation of a truncated VSX2 protein that lacks a complete DNA binding homeobox domain (amino acids 148–207) [39]. These variants are mostly associated with bilateral A/M and coloboma and are rarely associated with other eye deformities, like cataract and cone-rod dysfunction [24,28,40].

In families MA144 and MA193 we identified one novel and one known disease-causing homozygous *PXDN* variant, respectively. *PXDN* encodes a peroxidasin protein which is expressed in the epithelial layers of the cornea and lens, where it may provide structural support or serve as an antioxidant enzyme to protect the lens, cornea, and other developing eye structures from oxidative damage [27]. In family MA144, a high degree of intrafamilial phenotypic heterogeneity was observed where individual V:2 exhibits severe microphthalmia with corneal opacity, individual V:3 shows bilateral microphthalmia and unilateral corneal opacity, and individual V:4 was diagnosed with microphthalmia with anterior segment dysgenesis. In family MA193, we identified the previously reported 1bp deletion (c.2568del; p.(Cys857Alafs*5)) which was reported [27] in a Pakistani family with corneal opacity and cataract. In a Caucasian family, a different variant with the same protein effect (c.2569delT; p.(Cys857Alafs*5)) showed unilateral microphthalmia [41]. Contradictory to both previous studies, all affected individuals from family MA193 manifest bilateral anophthalmia, suggesting the variant is responsible for a more severe phenotype in this family. Several studies previously reported intra- and interfamilial phenotypic heterogeneity caused by *PXDN* variants even in monozygotic twins, which is in line with our findings [42]. Although our study is expanding the phenotypic spectrum of families carrying previously reported pathogenic variants in *FOXE3* and *PXDN* genes, the unavailability of OCT, ERG, or MRI for the patients is a limitation of our study in providing the complete phenotypic diversity.

Genome sequencing analysis in family MA144 revealed a novel deep intronic splice variant in intron 17 (c.3609-1307G>A) of *PXDN*. In silico splice site prediction tools predicted the activation of a pseudoexon (c.3609-1305_3609-1169) as a consequence of this variant. The expected pseudoexon insertion was evaluated by a minigene splice assay. The splice assay confirmed the activation of a pseudoexon, and an aberrantly spliced transcript could be observed that matched the in silico predictions. No wildtype transcript could be observed, suggesting that the variant causes a severe splice defect. The pseudoexon causes a change in reading frame, and the introduction of a premature stop codon. Therefore, the variant could be considered a loss-of-function variant. These findings are based on the severity of the mRNA defect, as observed in HEK293T cells, and RNA studies using patient-derived cells should be performed to completely assess the splice effect and the severity of the variant. Still, the splice assay did confirm a splice effect of the variant (c.3609-1305_3609-1169), p.Arg1203Serfs76*), and, therefore, the variant was classified as pathogenic and the phenotype of the family MA144 was considered genetically solved.

Previously, splice variants in *ALDH1A3, NAA10, RAX, TENM3*, and *VSX2* are already described to be associated with syndromic or non-syndromic A/M, but these were present either in exons or intron–exon junctions [28,43,44,45,46]. To the best of our knowledge, this is the first study reporting the association of a deep intronic splice variant with A/M, and the first splice-altering variant identified in *PXDN*. These findings emphasize the added value of genome sequencing as a diagnostic tool for A/M and the importance of incorporating deep intronic regions of the known A/M-associated genes in genetic analyses.

As extensively reviewed by Harding et al., the overall diagnostic solve rate of individuals affected by bilateral and severe A/M is 70%, which is reduced to only 10% when studying unilateral cases of A/M [8]. Although exome sequencing is an efficient and cost-effective method to perform genetic diagnostics, most genes associated with A/M are transcription factors and are GC-rich. Therefore, there is a chance that because of PCR bias and exon capture techniques, exome sequencing may fail to efficiently capture these GC-rich regions [47]. More recently, two studies explored the use of genome sequencing to increase diagnostic solve rates for A/M [48,49]. Neither of these studies focused on deep intronic regions of the genome due to limitations in their bioinformatic pipelines. In a study performed by Harding et al., an improved diagnostic rate of 33% was obtained when combining targeted panel testing with genome sequencing. The increased solve ratios were consistent for both unilateral and bilateral cases [48]. In a second study performed by Jackson et al., a diagnostic solve rate of 15.7% was achieved through genome sequencing for complex microphthalmia, anophthalmia, and coloboma patients [49]. We anticipate that when the assessment of deep intronic variants will also be incorporated, the diagnostic solve rates will improve even more. Although these previous findings and findings of the current study indicate that genome sequencing is a promising and effective diagnostic tool for A/M, considering the high sequencing costs, it is not feasible to provide genome sequencing to all patients. Hence, to make it cost-effective, in this study, we also used single gene *FOXE3* testing prior to genome sequencing to establish a genetic diagnosis. This approach led us to the solve rate of 100% in a relatively small cohort (seven families) exhibiting severe forms of bilateral A/M, suggesting that this is a cost-effective and feasible approach. Overall, this study highlights the usage of genome sequencing for the identification of coding and non-coding novel variants which eventually will lead towards better understanding of the complex inheritance pattern, associated comorbidities, and phenotypic variation among families affected with A/M.

## Figures and Tables

**Table 1:** Clinical features of affected individuals from seven families.

Clinical Feature	Family ID																			
	MA102			MA125		MA144			MA174					MA193		MA201			MA203	
	IV:2	IV:4	IV:5	IV:1	IV:2	V:2	V:3	V:4	IV:2	IV:3	IV:4	IV:6	IV:8	IV:1	IV:2	III:1	III:2	III:3	IV:2	IV:3
Phenotype	A	A	A	M	M	M	M	M	A	A	A	A	A	A	A	M	M	M	M	M
ASD	NA	NA	NA	-	+	-	-	+	NA	NA	NA	NA	NA	NA	NA	-	-	-	+	+
Visual acuity	NA	NA	NA	PL	PL	NLP	PL	PL	NA	NA	NA	NA	NA	NA	NA	NLP	NLP	PL	PL	PL
Corneal opacity	NA	NA	NA	+	+	+	RE	+	NA	NA	NA	NA	NA	NA	NA	+	+	+	+	+
Flat nasal bridge	+	+	+	-	-	+	+	+	+	+	+	+	+	+	+	+	+	+	-	-

All cases were diagnosed with recessively inherited congenital bilateral anophthalmia or microphthalmia. No facial dysmorphism, intellectual disability or developmental delays were observed in any of the affected individuals. A, anophthalmia; ASD, anterior segment dysgenesis; M, microphthalmia; NA, not applicable; NLP, no perception of light; PL, perception of light; RE, right eye; +, present; -, absent.

**Table 2:** In silico predictions for putative pathogenic variants identified.

Family ID	Gene	cDNA	Protein	gnomAD AF Total	gnomAD AF South Asian	CADD_PHRED	REVEL	SpliceAI	ACMG/AMP	Reference
MA102, MA125, MA203	*FOXE3*	c.720C>A	p.(Cys240*)	0.00001334 (Hom:0, Het:2)	0.0004148 (Hom:0, Het:2)	36	NA	NA	Pathogenic	Valleix et al., 2006 [26]
MA201	*FOXE3*	c.289A>G	p.(Ile97Val)	0.000006695 (Hom:0, Het:1)	0.0002126 (Hom:0, Het:1)	24.9	0.77	NA	Pathogenic	Ullah et al., 2016 [24]
MA144	*PXDN*	c.3609-1307G>A	p.Arg1203Serfs76*	0.000006571 (Hom:0, Het:1)	-	NA	NA	0.97 (AG)	Likely pathogenic	This study
MA174	*VSX2*	c.413_425del	p.(Ser138*)	-	-	NA	NA	NA	Pathogenic	This study
MA193	*PXDN*	c.2568del	p.(Cys857Alafs*5)	-	-	NA	NA	NA	Pathogenic	Khan et al., 2011 [27]

Candidate variants were identified in *FOXE3* (NM_012186.3), *PXDN* (NM_012293.3), and *VSX2* (NM_182894.3). All variants were found in a homozygous state in all affected members of the respective families. Thresholds for pathogenicity of the different in silico prediction tools: CADD_PHRED (≥15), REVEL (≥0.3), and spliceAI (≥0.2). ACMG/AMP, variant classification according to the American College of Medical Genetics and Genomics and Association for Molecular Pathology (ACMG/AMP) classification guidelines as described by Richards et al. [21]; AG, acceptor gain; CADD_PHRED, Combined Annotation Dependent Depletion PHRED score; cDNA, cDNA variant position based on the MANE select transcript; gnomAD AF South Asian, allele frequency in the South Asian population according to the gnomAD (v.3.1.2) database; GnomAD AF Total, allele frequency in the total population according to the gnomAD (v.3.1.2) database; Het, number of heterozygotes in the gnomAD database; Hom, number of homozygotes in the gnomAD database; NA, not applicable; SpliceAI, splice prediction delta score; -, absent; * termination codon.

## Data Availability

Data are available upon reasonable request.

## Supplementary Materials

The following supporting information can be downloaded at: https://www.mdpi.com/article/10.3390/genes14081573/s1, Table S1: Primer sequences; Table S2: Anophthalmia- and microphthalmia-associated genes as listed in OMIM; Figure S1: Sanger sequencing chromatograms for all the identified candidate variants in this study.
