# Supplementary material for: Combined Single Gene Testing and Genome Sequencing as an Effective Diagnostic Approach for Anophthalmia and Microphthalmia Patients
Source: Genes (Basel). 2023 Aug 1;14(8):1573. doi: 10.3390/genes14081573 (PMC10454697; doi:10.3390/genes14081573)
Supplement: Supplementary file 1 [file genes-14-01573-s001.zip › genes-2526506-supplementary.pdf]

Supplemetary Table S1: Primer sequences

Gene	Forward primer (5'-3')	Reverse primer (5'-3')
Primer sequences for targeted sequencing and segregation analysis		
FOXE3 (Exon 1)	GGGTACCGTGAACAGAGTAAG	TGTAGCAGGAGTTTGAGTCCAG
PXDN (Intron 17)	GTCCACACCTTTTCCCAGAG	GCTAGGTCCCTGGTAAAGAAG
VSX2 (Exon 2)	ACGAAATCTGTTCAAAACCTCCG	CTCATCCTGCTCAACCGGTG
PXDN (Exon 17)	GGAGTTCATGAGCAGCGAAG	GAATGGCTTCAACACCCCTC
Primer sequences for minigene splice assay		
PXDN (Intron 17)	TCCCAGATGATTCTCTCTTTTGC	AGCTCTAAGATGTCCCCTGG
ACTB (Exons 4-5)	ACTGGGACGACATGGAGAAG	TCTCAGCTGTGGTGGTGAAG
RHO (Exon 3-5)	CGGAGGTCAACAACGAGTCT	AGGTGTAGGGGATGGGAGAC

Supplementary Table S2: Anophthalmia- and microphthalmia-associated genes as listed in OMIM

Gene	OMIM	Gene	OMIM	Gene	OMIM	Gene	OMIM
ABCB6	605452	*ESCO2*	609353	*NF1*	613113	*SALL4*	607343
ALDH1A3	600463	*FAM111A*	615292	*NHS*	300457	*SCLT1*	611399
ALDH6A1	603178	*FANCA*	607139	*NUP188*	615587	*SEMA3E*	608166
ALX1	601527	*FANCD2*	613984	*OCLN*	602876	*SHH*	600725
ARHGAP6	300118	*FANCF*	613897	*OLFM2*	617492	*SIN3A*	607776
ATOH7	609875	*FANCL*	608111	*OTX2*	600037	*SIPA1L3*	616655
B3GALNT2	610194	*FKRP*	606596	*PAX2*	167409	*SIX3*	603714
BCOR	300485	*FKTN*	607440	*PAX6*	607108	*SIX6*	606326
BEST1	607854	*FNBP4*	615265	*PCYT1A*	123695	*SLC16A12*	611910
BMP4	112262	*FOXE3*	601094	*PDE6D*	602676	*SLC38A8*	615585
BRCA1	113705	*FREM1*	608944	*PITX3*	602669	*SMCHD1*	614982
BRCA2	600185	*FREM2*	608945	*PLK4*	605031	*SMG9*	613176
BRIP1	605882	*GDF3*	606522	*PNPT1*	610316	*SMOC1*	608488
C12orf57	615140	*GDF6*	601147	*POMGNT1*	606822	*SNX3*	605930
C2CD3	615944	*GJA1*	121014	*POMK*	615247	*SOX2*	184429
CAPN15	603267	*GJA8*	600897	*POMT1*	607423	*STRA6*	610745
CDKL5	300203	*GLI2*	165230	*POMT2*	607439	*TBC1D20*	611663
CENPF	600236	*GSC*	138890	*PQBP1*	300463	*TBC1D32*	615867
CEP120	613446	*GTF2H5*	608780	*PRMT7*	610087	*TBL1XR1*	608628
CHD7	608892	*HCCS*	300056	*PRR12*	616633	*TCTN2*	613846
CHUK	600664	*HDAC6*	300272	*PRSS56*	613858	*TENM3*	610083
CNTNAP1	602346	*HMGB3*	300193	*PTCH1*	601309	*TFAP2A*	107580
COL4A1	120130	*HMX1*	142992	*PUF60*	604819	*TKFC*	615844
COX14	614478	*HUWE1*	300697	*PXDN*	605158	*TMEM216*	613277
COX7B	300885	*INTS1*	611345	*RAB18*	602207	*TMTC3*	617218
CRPPA	614631	*INTU*	610621	*RAB3GAP1*	602536	*TMX3*	616102
CRYAA	123580	*KERA*	603288	*RAB3GAP2*	609275	*TOGARAM1*	617618
CRYBA4	123631	*KIF11*	148760	*RARB*	180220	*TUBB*	191130
CRYBB2	123620	*LMBRD2*	619490	*RAX*	601881	*TUBGCP4*	609610
DAG1	128239	*LRP5*	603506	*RBBP8*	604124	*VAX1*	604294
DOCK6	614194	*MAB21L2*	604357	*RBP4*	180250	*VCAN*	118661
DONSON	611428	*MAF*	177075	*RERE*	605226	*VSX2*	142993
DPYD	612779	*MAPRE2*	605789	*RHOA*	165390	*WDR37*	618586
ERCC1	126380	*MFRP*	606227	*RIPK4*	605706	*YAP1*	606608
ERCC3	133510	*MITF*	156845	*RPGRIP1L*	610937	*ZBTB20*	606025
ERCC5	133530	*NAA10*	300013	*RTTN*	610436	*ZEB2*	605802
ERCC6	609413	*NDUFB11*	300403	*RXYLT1*	605862		

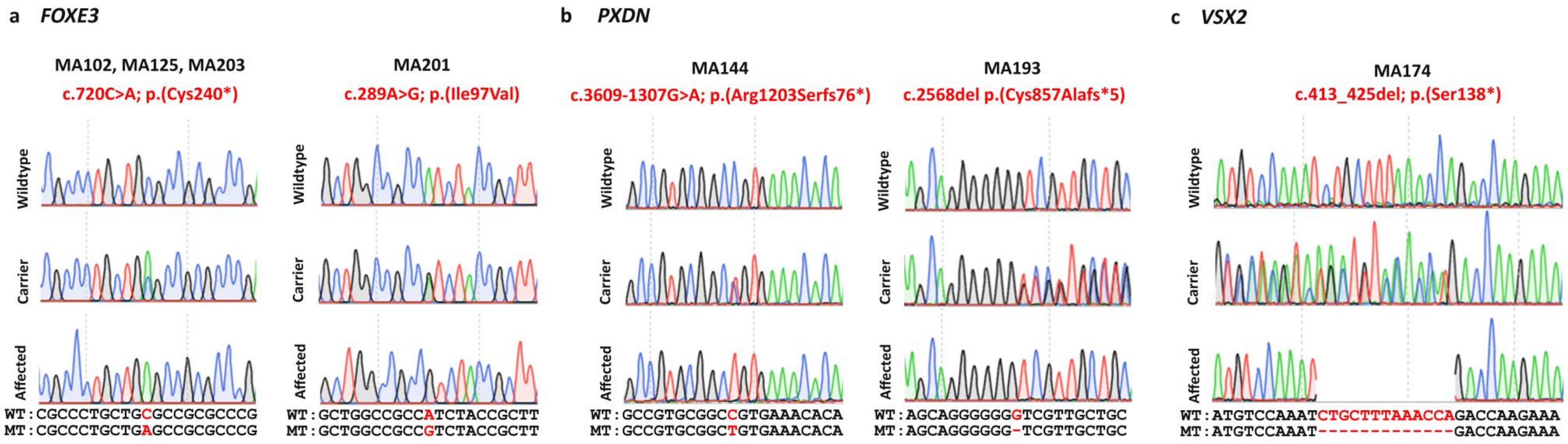

Supplemetary Figure S1: Sanger sequencing chromatograms for all the identified candidate variants in this study. Co-segregation of the candidate variant with the anophthalmia/microphthalmia phenotype was confirmed in all families. Complete segregation results can be found in Figure 1. For each variant, a representative chromatogram of a non-affected, carrier and an affected individual is provided. MT, mutated sequence; WT, wildtype sequence.
